# Excess folic acid disrupts placental endocrine function *in vitro*: a potential mechanism linking elevated folic acid exposure with gestational diabetes mellitus

**DOI:** 10.3389/fnut.2026.1813142

**Published:** 2026-05-28

**Authors:** Jessica M. Williamson, Anya L. Arthurs, Melanie D. Smith, Shalem Leemaqz, Alexander Phillips-Hughes, Dylan McCullough, Claire T. Roberts, Tanja Jankovic-Karasoulos

**Affiliations:** 1 Flinders Health and Medical Research Institute, College of Medicine and Public Health, Flinders University, Adelaide, SA, Australia; 2 Robinson Research Institute, School of Biomedicine, The University of Adelaide, North Terrace, Adelaide, SA, Australia

**Keywords:** folate, food fortification, periconceptional supplementation, placenta, pregnancy

## Abstract

**Background:**

High folic acid (FA; synthetic folate) intake is associated with increased risk of gestational diabetes mellitus (GDM), but the underlying mechanisms remain unknown. As the placenta is a key regulator of maternal glucose homeostasis, it represents a plausible target for FA-mediated effects.

**Objective:**

To determine how increased FA exposure modulates trophoblast function, placental folate receptor/transporter expression, and secretion of hormones that maintain maternal euglycaemia, providing a potential mechanism linking excess FA to GDM.

**Methods:**

Trophoblast cells (HTR-8/SVneo, JEG-3 and BeWo) and early- to mid-gestation human placental explants (*n* = 66) were cultured across a FA dose range representing acute physiological deficiency to excess (0 nM, 10 nM, 40 nM; 200 nM; 2000 nM). Trophoblast proliferation, migration and invasion were assessed in real time; placental explant proliferation and apoptosis by immunohistochemistry; folate receptor/transporter abundance by immunoblotting; and hormone secretions by enzyme-linked immunosorbent assays (ELISA). Statistical analyses were performed relative to 40 nM FA (physiological norm).

**Results:**

FA exposure produced a U-shaped response in trophoblast proliferation, migration, invasion, placental folate receptor (FOLR1) expression and human placental lactogen (hPL) secretion, where both acute deficiency and excess induced similar results relative to physiological norm. Notably, only excess FA significantly increased placental growth hormone (GH2) secretion (↑26%, *p* = 0.03), a hormone that promotes maternal insulin resistance.

**Conclusion:**

Human placental function and folate transport pathways are sensitive to FA exposure under conditions of deficiency and excess. Because folate deficiency is known to compromise pregnancy health, these findings suggest that excessive FA exposure may also disrupt placental function and perturb maternal and infant outcomes. The selective increase in GH2 under FA excess offers a plausible mechanism linking high FA exposure to increased GDM risk. Given widespread FA food fortification and increased use of high-dose prenatal supplements, the potential impact of FA excess on overall pregnancy metabolic health warrants urgent consideration.

## 1 Introduction

Periconceptional folate deficiency increases risk for neural tube defects (NTD). The World Health Organization (WHO) recommends daily supplementation with 400 μg folic acid (FA; synthetic folate) from preconception through 12 weeks’ gestation (1, 2). However, because not all pregnancies are planned, and the neural tube closes by ~4 weeks after conception (6 weeks gestation), many women remain at risk of folate deficiency and NTDs (3). To address this, more than 90 countries have introduced mandatory FA food fortification alongside recommendations for periconceptional supplementation (4). These initiatives have successfully increased serum folate (5–7) and red cell folate (7, 8) concentrations, and reduced NTD prevalence compared with pre-fortification baselines (9).

Combined intake from FA fortification and/or prolonged or high-dose supplementation, means that many pregnant women now exceed recommended FA intakes and, in some cases, the established upper tolerable limit of 1,000 μg/day (10, 11). This limit is based on the potential to mask vitamin B12 deficiency rather than on adverse pregnancy outcomes, as no pregnancy-specific upper limit for FA intake or folate status currently exists. Elevated maternal red cell folate concentrations above the upper clinical reference range have become far more prevalent than deficiency (12) and detectable unmetabolised FA in circulation (13–20) are increasingly reported. While the adverse effects of folate deficiency on pregnancy outcomes are well established (4, 21–23), the consequences of excessive FA exposure and unmetabolized FA remain comparatively understudied and poorly defined.

Emerging evidence links both high FA intake and elevated maternal folate status with adverse metabolic outcomes in pregnancy (24–29), including increased risk of gestational diabetes mellitus (GDM) (30–32) but the mechanism remains poorly understood. Importantly, no relationship between folic acid and type 2 diabetes mellitus has been documented, suggesting that a pregnancy-specific mechanism underpins this association.

The placenta is a transient organ of fetal origin that plays a central role in maternal-fetal exchange and orchestrates maternal adaptations to pregnancy (33). It is highly responsive to environmental and nutritional cues throughout gestation, and demonstrates a remarkable capacity for dynamic adaptation (34, 35), which makes it a plausible mechanistic target of FA action. The placenta expresses a network of folate/FA transport proteins - folate receptor 1 (FOLR1), proton-coupled folate transporter (PCFT) and reduced folate carrier (RFC) (36, 37) - that mediate transfer of both synthetic FA and natural folate forms from maternal to fetal circulation (38, 39). Notably, the placenta has ~ 14-fold higher binding affinity for FA than for natural folate (40). Once internalised, FA enters one-carbon metabolism, which supports nucleotide synthesis, cellular repair, redox balance, and methylation reactions essential for epigenetic regulation (41, 42). Excess FA supply therefore has the potential to alter trophoblast proliferation or invasion (processes critical for establishing maternal-fetal exchange), and to influence transcriptional pathways, including endocrine pathways that govern maternal metabolic adaptations.

Placental hormones play a central role in coordinating maternal metabolic adaptations to pregnancy (43–46). Placental growth hormone variant (GH2) promotes systemic insulin resistance to ensure adequate glucose supply to the fetus, whereas human placental lactogen (hPL) and pituitary prolactin (PRL) primarily stimulate maternal insulin secretion to maintain euglycaemia (47–49). Other placental hormones, including progesterone (P4), pregnancy-associated plasma protein A (PAPP-A) and human chorionic gonadotropin (hCG), have also been implicated in maternal metabolic regulation, although their contributions appear less direct and are not as well characterised as those of GH2 and hPL (50–52). A recent study demonstrated that FA food fortification is associated with elevated circulating GH2 and hPL, but not PRL, in both healthy and GDM-complicated pregnancies (53), suggesting that high FA exposure may disrupt placental endocrine balance but causal evidence is lacking.

Although observational studies indicate that FA exposure may influence aspects of placental function, the specific pathways through which FA affects early trophoblast behaviour and endocrine function remain poorly defined. In particular, dose-dependent effects and the integrated consequences for FA transport, trophoblast proliferation and hormone secretion have not been examined together. This study therefore investigated how increasing FA exposure affects multiple facets of placental function *in vitro*. We hypothesized that higher FA doses would alter expression of folate receptors and/or transporters, promote trophoblast proliferation, and modify secretion of key placental hormones, thereby providing a potential mechanistic link between FA exposure and GDM.

## 2 Methods

### 2.1 Human placental cell lines

All cell lines were purchased from the American Type Culture Collection. HTR-8/SVneo extravillous trophoblast cells were cultured in FA-depleted RPMI-1640 supplemented with 5% fetal bovine serum (Sigma), 1% antimycotic-antibiotic (Sigma), while BeWo and JEG-3 cells were cultured in FA-depleted Dulbecco′s Modified Eagle′s Medium (DMEM, Sigma Aldrich), supplemented with 3.7 g/L sodium bicarbonate (Gibco), 0.584 g/L L-glutamine (Gibco), 5% fetal bovine serum (Sigma) and 1% antimycotic-antibiotic (Sigma), at 37 °C, 5% CO_2_ and 20% O_2_.

### 2.2 Proliferation assay

Cell proliferation was assessed using the xCELLigence Real-Time Cell Analysis Dual Plate (RCTA DP; Agilent). Seeding densities were as follows: 5×10^3^ cells/well for HTR-8/SVneo and JEG-3 cells, and 6.5×10^3^ cells/well for BeWo cells. At 6 h post-seeding, FA supplemented media was added to each well to constitute final FA concentrations of 0 nM, 10 nM, 40 nM, 200 nM and 2000 nM. Although no additional FA was added to the 0 nM treatment, the actual FA concentration was ~2 nM due to FA present in the serum. E-plates were placed into the Real-Time Cell Analysis Dual Plate (RTCA DP) cradle, and cell index measurements were recorded every 15 min over a 72-h period using the RTCA software (Agilent, Santa Clara, CA, USA).

### 2.3 Migration assay

Cell migration was assessed using the IncuCyte® Live-Cell Analysis System (Sartorius, Göttingen, Germany). HTR-8/SVneo cells were chosen for migration and invasion assays because they are derived from primary first-trimester extravillous trophoblasts. As a transformed extravillous trophoblastic lineage, they retain the migratory and invasive characteristics of their progenitor cells, which migrate into the maternal decidua to establish placental anchorage *in vivo*. HTR-8/SVneo cells (1.5×104 cells/well) were seeded in 96-well IncuCyte® ImageLock plates (Sartorius). 24 h-post seeding uniform scratch wounds were created using the WoundMaker™ tool (Sartorius). Images were captured at 2-h intervals over 24 h. Relative wound density was calculated using the image analysis algorithm provided by Sartorius Incucyte® Scratch Wound Analysis Software Module, as defined in the manufacturer’s protocol (54, 55).

### 2.4 Invasion assay

Cell invasion was measured using the xCELLigence CIM-Plate 16 (Agilent,) pre-coated with Matrigel® Matrix (Corning). HTR-8/SVneo cells (3×10^4^) were seeded in the upper chamber of each well. CIM-plates were placed into the RTCA DP cradle, and cell index measurements were recorded every 30 min for 24 h using the RTCA software.

### 2.5 Human placental tissue collection and explant culture

Ethical approval was granted by The Queen Elizabeth Hospital Human Research Ethics Committee (HREC/16/TQEH/33). First (n = 30; 6–12 weeks’ gestation, calculated from onset of last menstrual period) and second (n = 36; 13–16 weeks’ gestation) trimester human placentae were collected with written informed consent from women who underwent elective terminations of otherwise normal pregnancies at the Pregnancy Advisory Centre, Adelaide, South Australia, between 2022 and 2024. Sections of villous tissue (10–15 mg) were dissected and plated in 48-well plates on Growth Factor Reduced Matrigel® Matrix Basement Membrane (Corning). Cultures were maintained at 37 °C, in 5% CO_2_ and physiologically relevant O_2_ for each gestation: 1% O_2_ for first trimester and 8% O_2_ for second trimester explants.

### 2.6 Folic acid treatment

Explants were initially cultured for 48 h in FA-depleted media (DMEM; Sigma Aldrich) supplemented with 3.7 g/L sodium bicarbonate (Gibco), 0.584 g/L L-glutamine (Gibco), 5% Fetal Bovine Serum (Sigma) and 1% antimycotic-antibiotic, adjusted to pH 7.0 (56). Following the initial incubation period, explants were treated with media supplemented with FA (Sigma Aldrich) for 72 h at the following concentrations: 0 nM (severe acute deficiency; no added FA, however, final folate/FA concentration was ~2 nM due to residual FA from FBS), 10 nM (deficient; reflecting the WHO folate deficiency threshold); 40 nM (adequate; upper reference limit for serum folate as per the Royal College of Pathologists of Australasia [RCPA] – note WHO does not define a full reference range, only cut-off value to identify deficiency or adequacy in the context of public health interventions); 200 nM (elevated); and 2000 nM (excess). Both 200 nM and 2000 nM treatments were based on maternal serum folate levels observed post-fortification in the STOP cohort (12)At the end of this time, explants were weighed for normalisation, and culture media collected for measurement of placental hormone concentrations. Explant tissue was either snap-frozen in liquid nitrogen and stored at −80 °C for subsequent immunoblotting, or formalin-fixed, paraffin-embedded for subsequent immunohistochemistry.

### 2.7 Enzyme-linked immunosorbent assays (ELISA)

Placental hormone concentrations secreted from *in vitro* treated placentae were measured using ELISA as per manufacturer’s instructions. Human placental lactogen (hPL) and progesterone ELISA kits were purchased from ALPCO (Salem, NH, USA). Growth hormone variant (GH2), Pappalysin-1 (PAPP-A) and β-human Chorionic Gonadotropin (β-hCG) kits were purchased from FineTest (Cambridge, UK). Absorbance was measured at 450 nm using SpectraMax iD5 plate reader. Final values were normalised to total placental explant weight (mg). Hormone concentrations were analysed using linear regression with post-hoc Dunnett’s test, comparing each treatment group to 40 nM FA treatment, which was considered the physiological reference. Statistical analyses were performed using R version 4.2.1.

### 2.8 Immunoblotting

Total placental protein was extracted, and proteins were separated using 4–20% protein gels (BioRad). Membranes were blocked overnight at 4 °C in 5% (w/v) skim milk powder before being incubated with primary antibodies (Table 1) for 1.5 h at room temperature, under agitation. Following primary antibody incubation, membranes were incubated with secondary antibodies (for 1 h at room temperature with agitation). Membranes were visualized using ImageQuant LAS 4000 biomolecular imager. Protein band intensities were calculated using protein densitometry using ImageJ software (57). Protein expression was normalised to total protein, visualized with Amido Black (58, 59). Statistical analysis was performed using one-way ANOVA, comparing FA treatment groups relative to 40 nM [upper reference range for serum folate, recommended by Royal College of Pathologists Australia (60)].

**Table 1:** Primary antibodies used for immunoblotting and immunohistochemistry.

Target	Supplier	Cat No.	Dilution factor	
			Immunoblotting	Immunohistochemistry
Folate receptor 1 (FOLR1)	Invitrogen	PA5-24186	1:1000	-
Proton-coupled folate transporter (PCFT)	Abcam	Ab25134	1:1000	-
Reduced folate carrier (RFC)	Santa Cruz Biotechnology	sc-390948	1:1000	-
Proliferating cell nuclear antigen (PCNA)	Atlas Antibodies	HPA030522	-	1:50
Cleaved Caspase 3	Cell Signaling Technology	#9661	-	1:50

### 2.9 Immunohistochemistry

Formalin-fixed, paraffin-embedded placental tissue sections (5 μM) were mounted onto Knittel StarFrost advanced adhesive microscope slides (ProSciTech). Sections were dewaxed, rehydrated and subjected to sodium citrate antigen retrieval. Tissue sections were incubated with primary antibodies (Table 1) for 1 h at 4 °C, followed by incubation with the appropriate secondary antibodies for 1 h at room temperature, and then with streptavidin-horseradish peroxidase (Streptavidin-HRP) for a further 1 h at room temperature. Diaminobenzidine (DAB) was applied for 15 min at room temperature, protected from light, and sections were counterstained with haematoxylin. Slides were rinsed, dehydrated and scanned using the VS200 slide scanner (Olympus, Tokyo, Japan). Images were viewed using the OlyVIA (Olympus) visual imaging program. For quantitative analysis of immunohistochemically stained tissue sections, eight fields of view were randomly selected per section. For quantification of proliferating cell nuclear antigen (PCNA) and cleaved caspase 3, positively stained cells were counted and reported as a percentage of total nuclei. Statistical analysis was performed using one-way ANOVA, with comparisons made relative to the 40 nM FA condition.

## 3 Results

### 3.1 Folic acid altered real-time proliferation kinetics in human trophoblast cell lines

**Figure 1:**
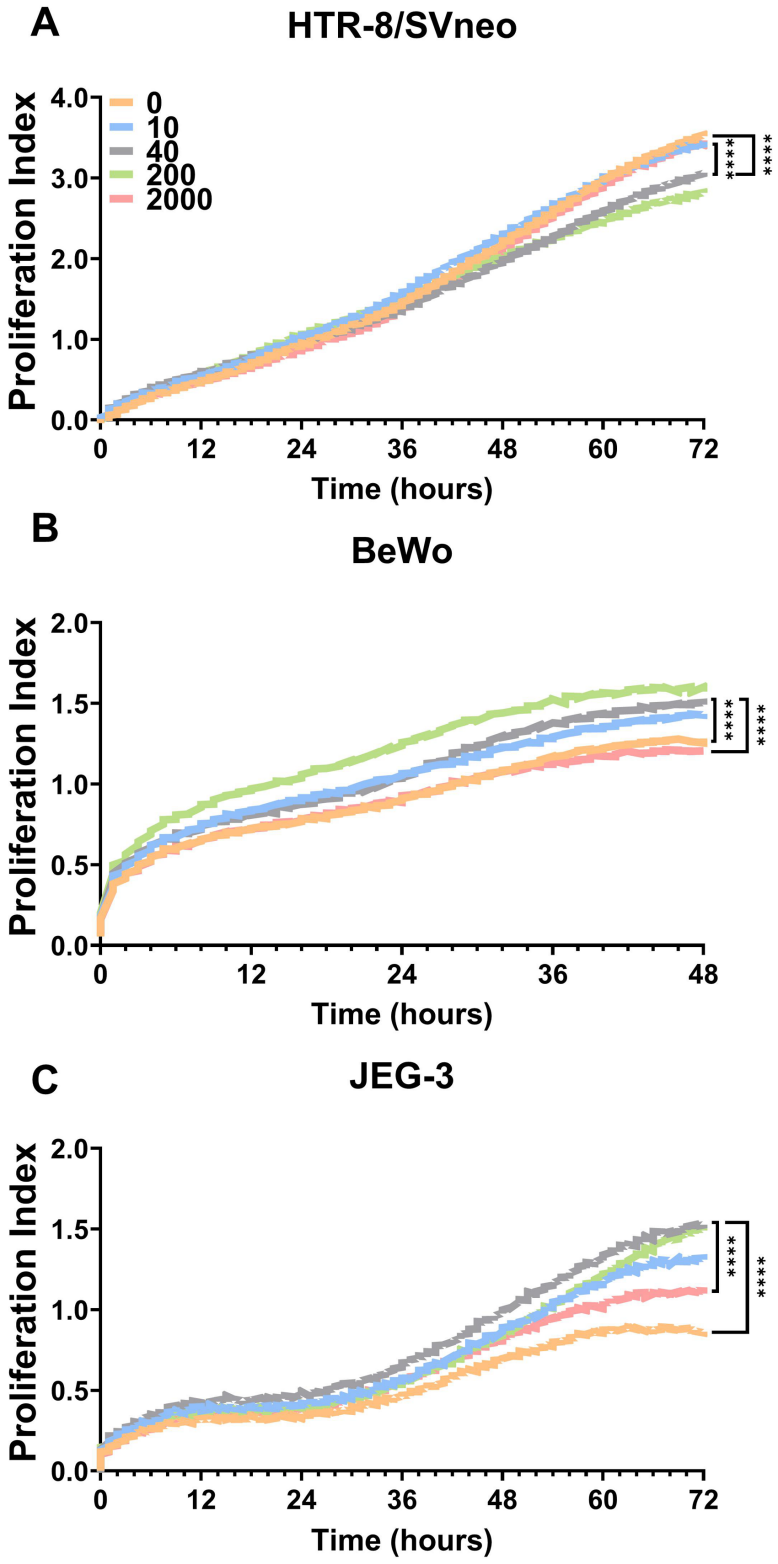
Effects of FA exposure on trophoblast cell proliferation in **(A)** HTR-8/SVneo, **(B)** BeWo, and **(C)** JEG-3 cells. Data are presented as mean cell index (*n* = 3–4) and analyzed by one-way ANOVA with Bonferroni *post hoc* comparisons against the 40 nM reference concentration (representing physiological norm). Colors indicate FA treatment dose: orange indicates 0 nM, blue indicates 10 nM, grey indicates 40 nM, green indicates 200 nM and pink indicates 2000 nM. Asterisks denote significance: ****p < 0.0001.

Trophoblast proliferation exhibited a non-linear response to FA, in a cell-type-specific manner that differs between extravillous and villous cytotrophoblast-like cell types (Figure 1). In HTR-8/SVneo extravillous cells both severe deficiency and excess increased proliferation. Conversely in villous cytotrophoblast-like BeWo and JEG-3 cells, both severe folic acid deficiency and excess decreased proliferation.

Using 40 nM FA as the reference dose, extravillous HTR-8/SVneo cells showed significantly higher proliferation at both deficient and excess FA concentrations across 72 h in culture: +7.67% at 0 nM (*p* < 0.0001), +11.37% at 10 nM (*p* < 0.0001), and +5.12% at 2000 nM (*p* < 0.0001) compared to 40 nM cultures. Proliferation at 200 nM did not differ from the reference (*p* > 0.9999) (Figure 1).

In BeWo villous cytotrophoblast-like cells, relative to 40 nM, both deficient and excess FA significantly reduced proliferation by 14.97% at 0 nM (*p* < 0.0001), 5% at 10 nM (*p* < 0.0001) and 16.39% at 2000 nM (*p* = 0.0002), while 200 nM FA resulted in increased proliferation (12.59%, *p* = 0.0083) relative to 40 nM (Figure 1).

In JEG-3 cells the highest proliferation index was observed in response to 40 nM FA. Relative to 40 nM FA treatment, all other doses significantly decreased proliferation with the largest effects observed in response to 0 nM and 2000 nM treatments. Relative reductions were: −38.52% at 0 nM (*p* < 0.0001), −12.42% at 10 nM (*p* = 0.007), −11.25% at 200 nM (*p* = 0.0176), and −21.87% at 2000 nM (*p* < 0.0001) (Figure 1).

### 3.2 Folic acid altered migratory capacity of HTR-8/SVneo cells

**Figure 2:**
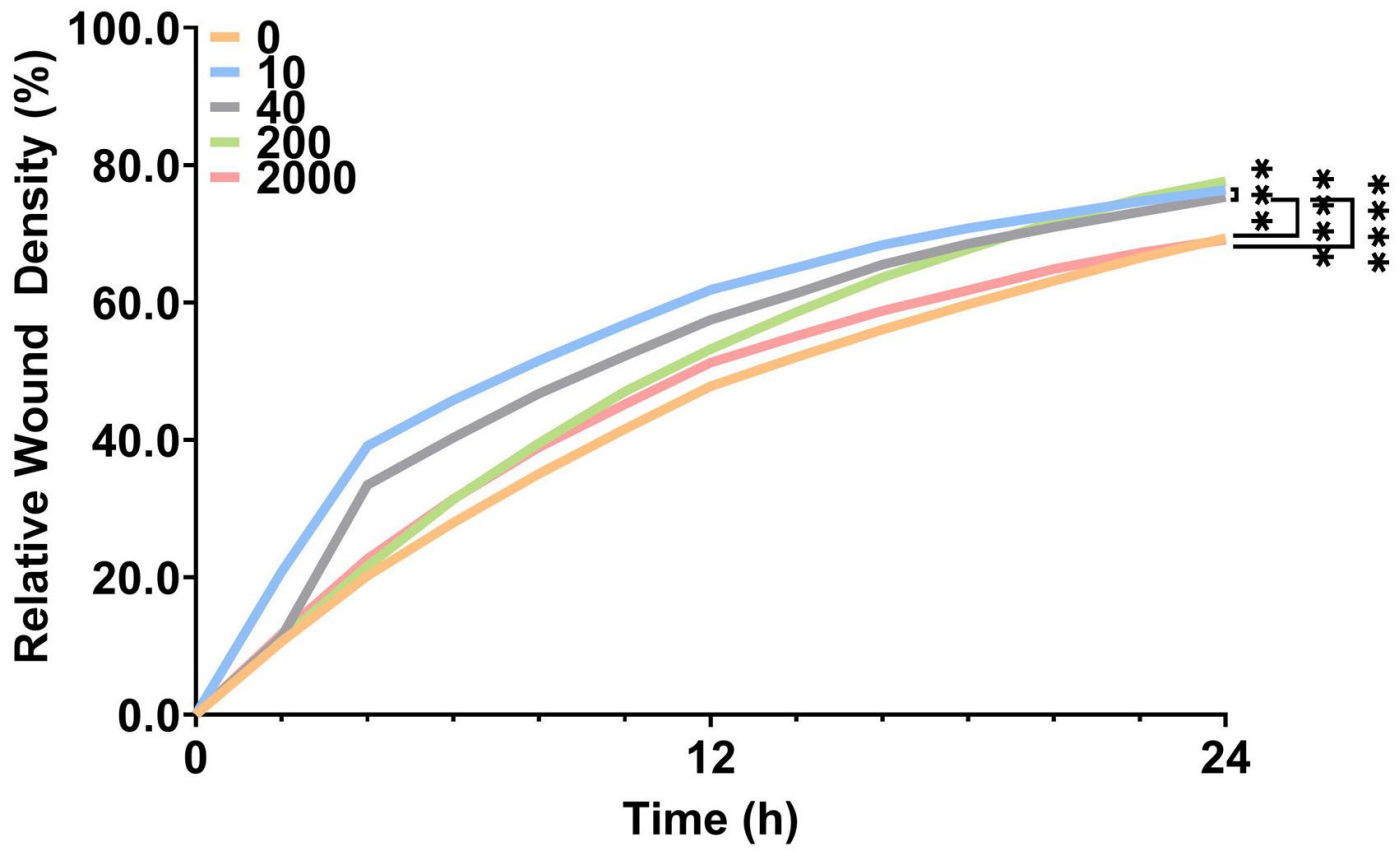
Effects of FA exposure on cell migration in HTR-8/SVneo cells. Data represent mean from three biological replicates. One-way ANOVA with Bonferroni *post hoc* test, comparing each treatment to the reference concentration of 40 nM that represents physiological norm. Colours indicate FA treatment dose: orange indicates 0 nM, blue indicates 10 nM, grey indicates 40 nM, green indicates 200 nM, and pink indicates 2000 nM. Asterisks denote significance: *** *p* < 0.001, **** *p* < 0.0001.

FA modulated migration of the extravillous trophoblast cell line HTR-8/SVneo *in vitro* (Figure 2). Relative to 40 nM reference concentration, migration was significantly reduced by 17.63% at 0 nM FA (*p* < 0.0001) and by 12.70% at 2000 nM FA (*p* < 0.0001). In contrast, a modest but significant increase of 6.99% was measured in response to 10 nM FA (*p* < 0.0001). Migration at 200 nM did not differ significantly from 40 nM reference (*p* = 0.1272).

### 3.3 Folic acid altered the invasive capacity of HTR-8/SVneo cells

**Figure 3:**
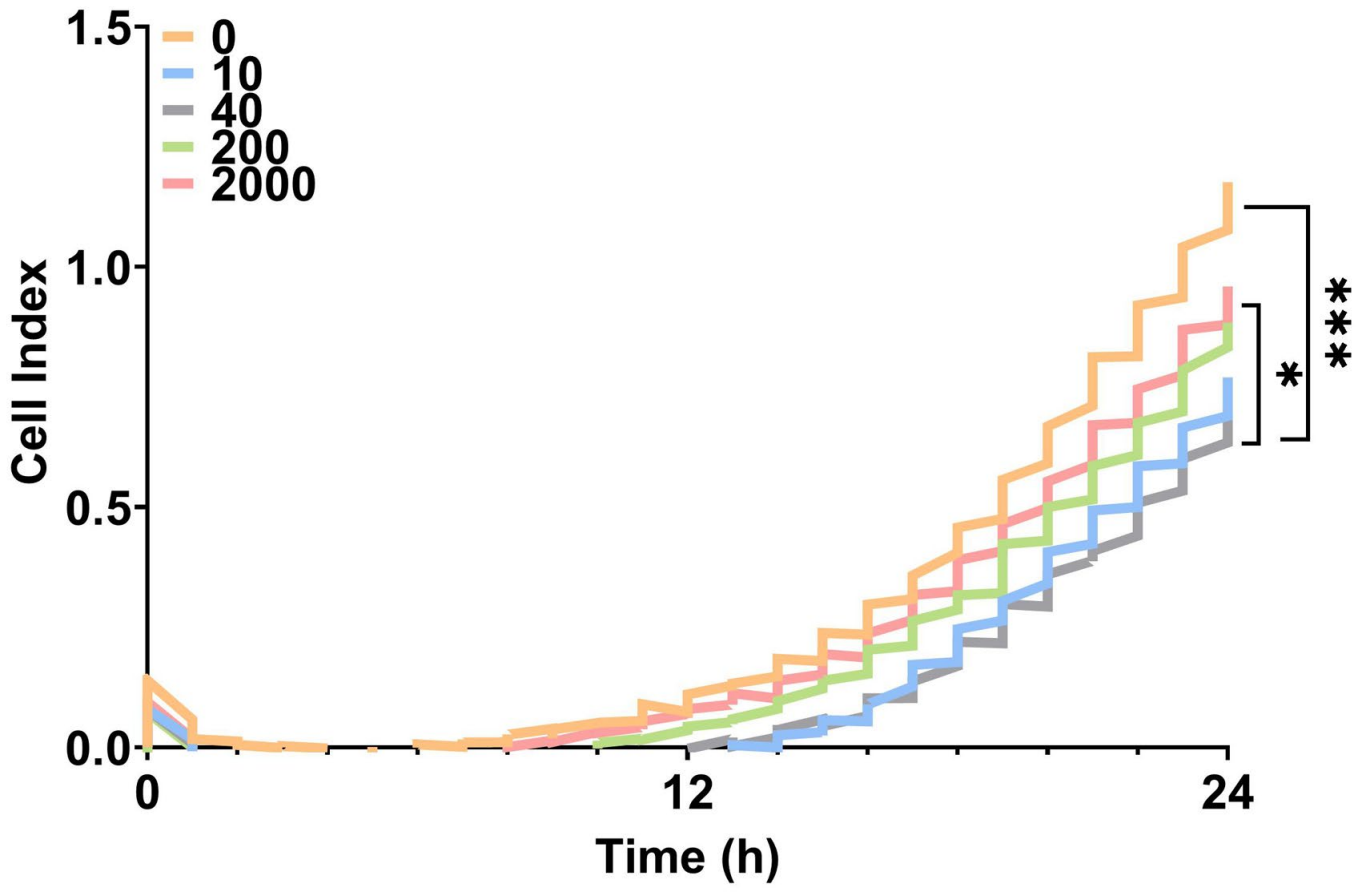
Effect of FA exposure on cell invasion in HTR-8/SVneo cells. Data represent mean from three biological replicates. One-way ANOVA with Bonferroni *post hoc* test, comparing each treatment to the reference concentration of 40 nM that represents physiological norm. Colors indicate FA treatment dose: orange indicates 0 nM, blue indicates 10 nM, grey indicates 40 nM, green indicates 200 nM, and pink indicates 2000 nM. Asterisks denote significance: * *p* < 0.05, *** *p* < 0.001.

FA modified invasive capacity of HTR-8/SVneo cells *in vitro* (Figure 3). Invasion was lowest at the physiological reference concentration of 40 nM. Relative to this reference, invasion increased significantly by 83.95% at 0 nM FA (*p* = 0.0002) and by 131.33% at 2000 nM FA (*p* = 0.0379). No significant changes in invasion were observed with 10 nM or 200 nM treatments.

### 3.4 Folic acid exposure did not alter placental explant proliferation or apoptosis indices

**Figure 4:**
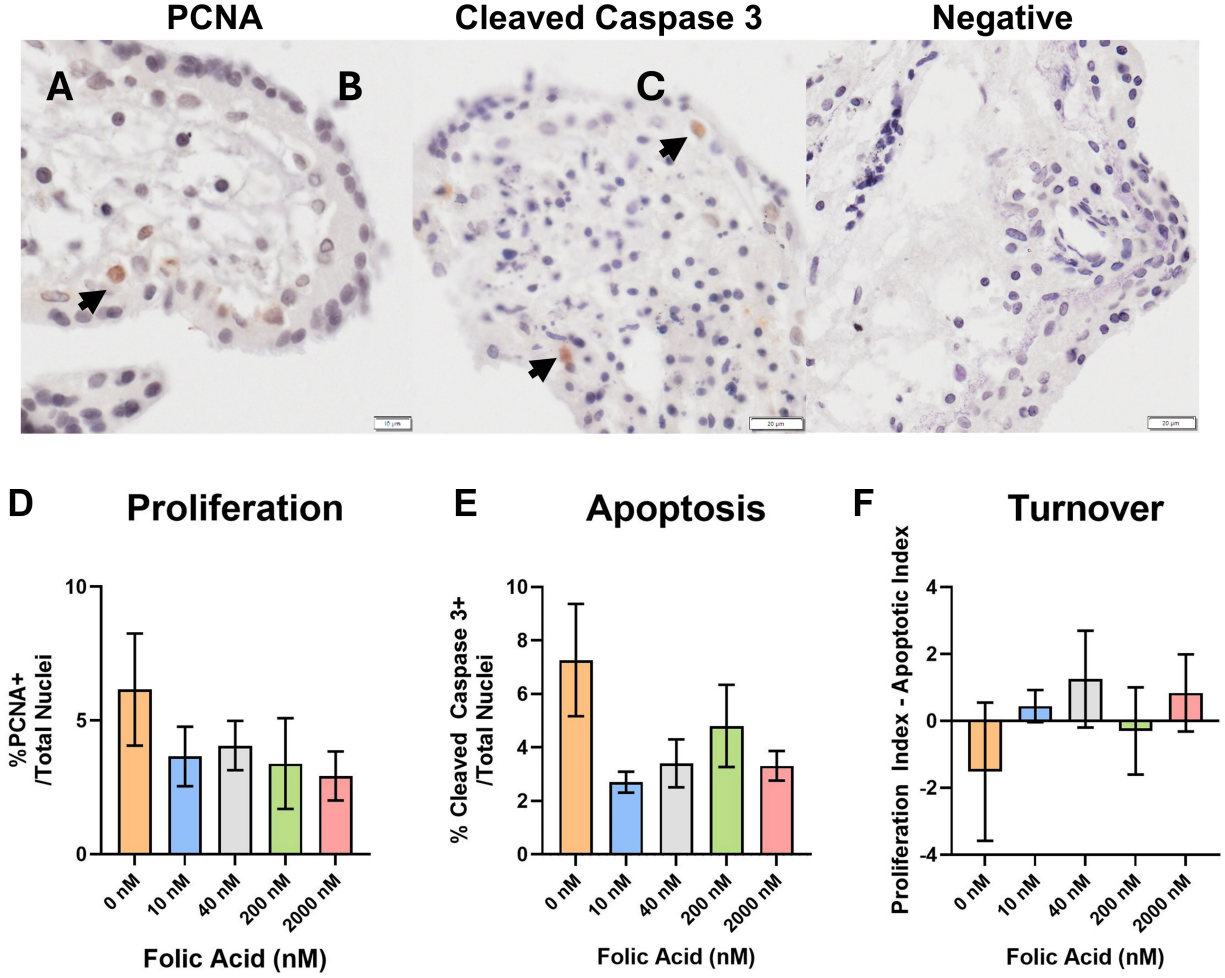
The effect of FA exposure on proliferation and apoptosis in first and second trimester human placental explants. Representative immunohistochemical images of **(A)** PCNA and **(B)** cleaved caspase 3 staining in formalin-fixed, paraffin-embedded placental explants (*n* = 6–8 per treatment) and quantification of **(C)** PCNA-positive nuclei as a percentage of total nuclei, **(D)** cleaved caspase 3-positive nuclei as a percentage of total nuclei, and **(E)** cellular turnover, calculated as the difference between PCNA-positive and cleaved caspase 3-positive nuclei (expressed as a percentage of total nuclei). Statistical analysis included one-way ANOVA with Bonferroni *post hoc*, with comparisons made relative to 40 nM FA treatment. FA treatment dose: orange indicates 0 nM, blue indicates 10 nM, grey indicates 40 nM, green indicates 200 nM, and pink indicates 2000 nM.

Cell proliferation in human placental explants was not significantly different between treatment groups, as assessed by proliferating cell nuclear antigen (PCNA) positivity in tissue sections (Figure 4). No significant differences in apoptosis were observed, as measured by caspase 3 positivity, in response to any FA treatment. Cellular turnover, calculated as the percentage of PCNA+ nuclei minus the percentage of cleaved caspase 3-positive nuclei, was also not significantly different across treatment groups (Figure 4).

### 3.5 Folic acid exposure altered the abundance of placental folate receptor 1 (FOLR1)

**Figure 5:**
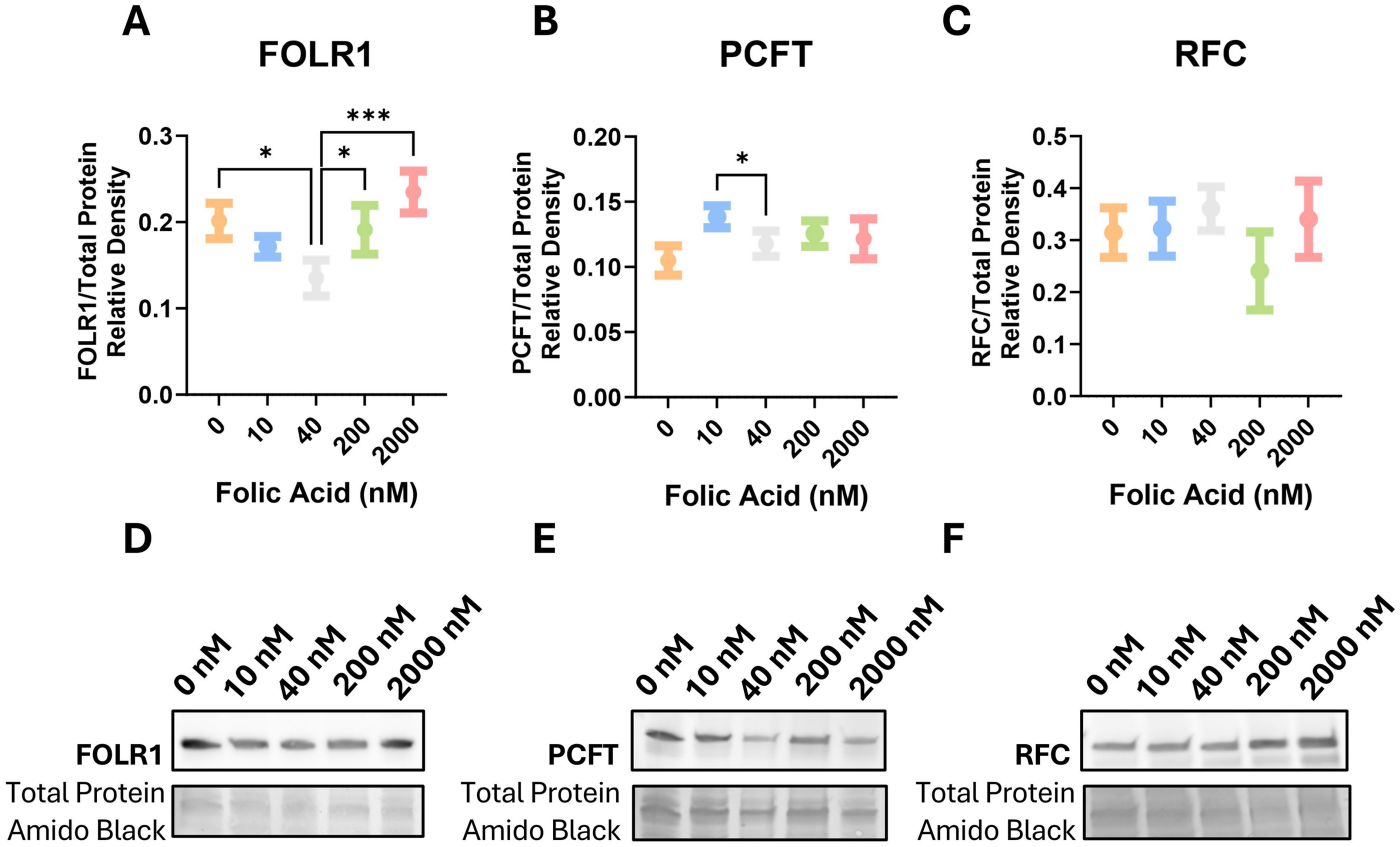
FA regulation of placental folate receptors and transporters. Relative protein expression of **(A)** folate receptor 1 (FOLR1), **(B)** proton-coupled folate transporter (PCFT), and **(C)** reduced folate carrier (RFC) (*n* = 8–12) in FA-treated placental explants. Representative immunoblots of **(D)** FOLR1, **(E)** PCFT, and **(F)** RFC. Statistical analysis included one-way ANOVA with comparison to the 40 nM group. Colors indicate FA treatment dose: orange indicates 0 nM, blue indicates 10 nM, grey indicates 40 nM, green indicates 200 nM, and pink indicates 2000 nM. Asterisks denote significance: *p < 0.05, ***p < 0.001.

FA exposure significantly altered FOLR1 and PCFT protein levels in human placental explants (Figure 5). Relative to 40 nM reference dose, FOLR1 abundance showed a non-linear response, with significant increases of 39.22% at 0 nM (*p* = 0.04), 34.24% at 200 nM (*p* = 0.03) and 53.77% at 2000 nM (*p* = 0.0004). In contrast, PCFT abundance increased significantly only in response to 10 nM FA (16.15%; *p* = 0.02). RFC protein levels were not significantly altered by any FA concentration *in vitro*.

### 3.6 Folic acid exposure altered placental secretion of GH2 and hPL, but not hCG, PAPP-A nor progesterone

**Figure 6:**
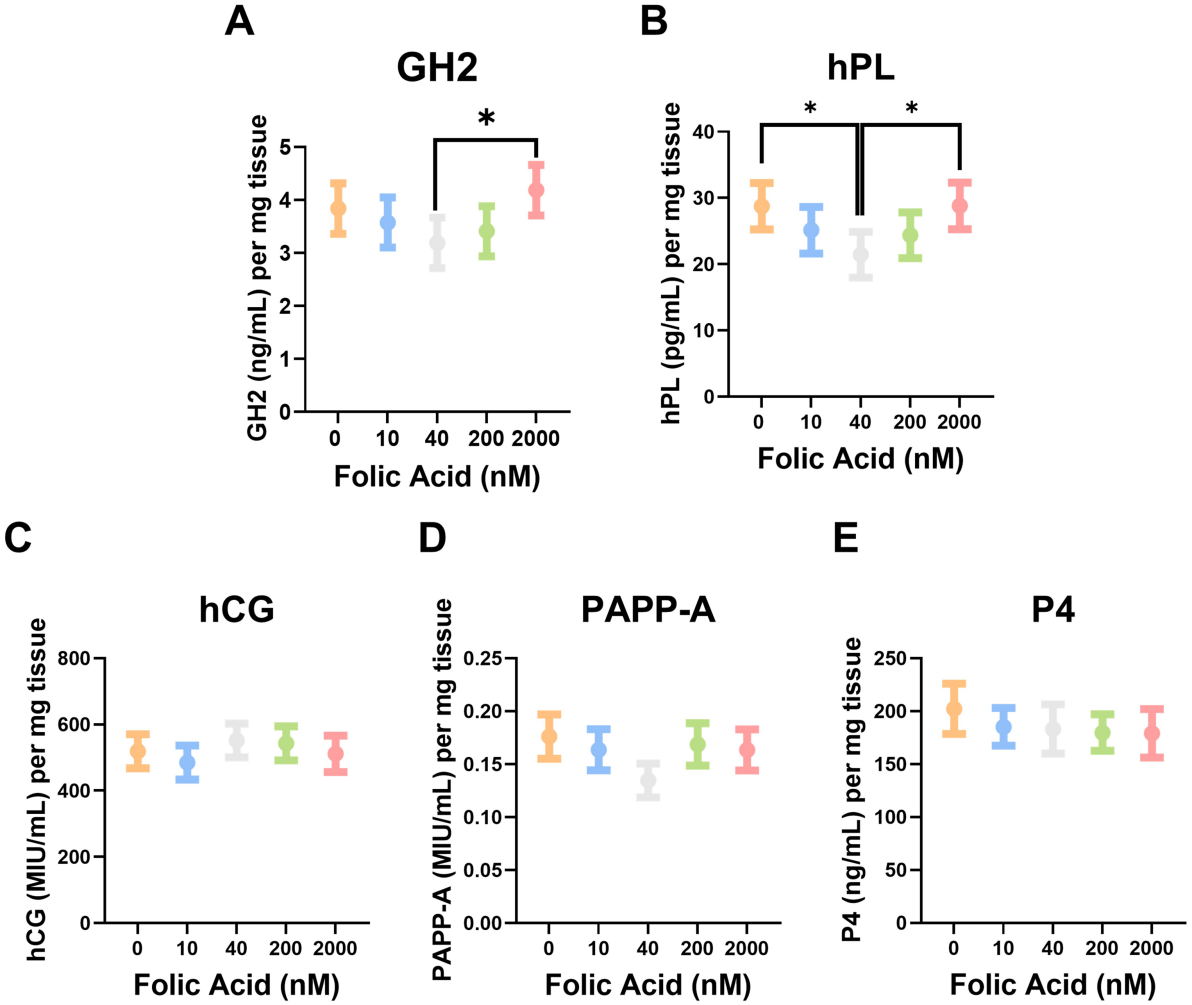
FA regulation of placental hormone secretion: **(A)** Placental growth hormone (GH2), **(B)** human placental lactogen (hPL), **(C)** human chorionic gonadotropin (hCG), **(D)** pregnancy associated plasma protein A (PAPP-A), and **(E)** progesterone (P4) in first and second trimester human placental explants (*n* = 66). Data are presented as estimated marginal mean ± SEM. Linear regression with post hoc Dunnett’s test relative to the 40 nM FA treatment. Colors indicate FA treatment dose: orange indicates 0 nM, blue indicates 10 nM, grey indicates 40 nM, green indicates 200 nM, and pink indicates 2000 nM. Asterisks denote significance: *p < 0.05.

Relative to the 40 nM reference, GH2 secretion increased significantly only in response to 2000 nM FA, rising by 26% (mean difference: 0.99; CI: 0.06–1.93, *p* = 0.03). In contrast, hPL secretion demonstrated a non-linear response, with significant increases in response to both 0 nM FA (24%; mean difference: 7.31 CI: 0.59–14.03, *p* = 0.03) and 2000 nM FA (29%; mean difference: 7.37 CI: 0.63, 14.10, *p* = 0.02) (Figure 6). FA did not significantly affect secretion of hCG, PAPP-A nor progesterone.

## 4 Discussion

While decades of research have established folate deficiency as a major concern in pregnancy (61, 62), the effects of excess exposure to FA, on pregnancy health remain comparatively understudied, despite global food FA fortification and frequent over-supplementation in many populations. Importantly, observational studies increasingly associate excessive FA intake and high maternal folate status with greater risk of gestational diabetes, highlighting the need to understand the mechanistic pathways through which FA may influence placental and metabolic function. In the present study, the effects of FA on placental function were investigated using complementary *in vitro* models. These included human trophoblast cell lines and first- and second-trimester human placental explants that preserve three-dimensional architecture and distinct cellular compartments, importantly with an intact syncytiotrophoblast layer - the principal site of placental endocrine activity that drives maternal metabolic adaptations. Across these models, we demonstrate that both folate deficiency and excess can alter trophoblast proliferation, migration and invasion, regulate placental folate receptor/transporters, and modify endocrine function. Notably, FOLR1 and hPL exhibited U-shaped responses to FA, with increased abundance under both deficient and excess conditions, relative to physiological norm. In contrast, GH2 secretion was upregulated *only* under FA excess, indicating a selective endocrine sensitivity to FA excess. Together, these findings provide evidence that both deficiency and excess disrupt placental behaviour, FA metabolism, and hormone secretion, and identify a plausible mechanistic pathway through which excessive FA intake may perturb placental endocrine signals that regulate maternal insulin resistance, providing a potential link between high FA exposure and increased GDM risk.

### 4.1 Effects of FA on trophoblast proliferation, migration and invasion

This study used several human trophoblast cell lines to investigate dynamic effects of FA on cellular function. HTR-8/SVneo cells were used as a model of extravillous trophoblast cells, the placental cellular subtype engaged in migration and invasion (63), while BeWo and JEG-3 cells were used to represent villous cytotrophoblast-like proliferative responses. Real-time proliferation analysis revealed a non-linear, cell-type-specific response to FA across all three trophoblast models.

In HTR-8/SVneo cells, proliferation followed a parabolic pattern, with significant increases under both severe deficiency (0 nM FA) and excess (2000 nM) (Figure 1). Ahmed et al. (56) previously reported increased proliferation in response to FA excess (2000 ng/mL; approximately 4,532 nM) but no change in response to FA-deficient conditions (2 ng/mL; approximately 4.5 nM). In contrast, our findings demonstrate increased proliferation in response to both severe (0 nM) and mild (10 nM) deficiencies. Cytotrophoblast-like cell types, BeWo and JEG-3 data, also exhibited parabolic patterns, but in contrast to HTR-8/SVneo cells, severe deficiency and excess significantly reduced proliferation relative to physiological norm. This contrasts with Ahmed et al. (56), who reported no differences in BeWo proliferation after 48 h of treatment. A likely explanation lies in methodological differences: real-time proliferation analysis, as used in our study, provides greater sensitivity to detect dynamic and time-dependent changes in cell proliferation. In our hands, conventional endpoint assays at fixed timepoints (data not shown) did not detect significant differences between treatment groups, highlighting the limitations of static measurements and suggesting that reliance on categorical timepoints may account for some of the inconsistencies between studies. Moussa et al. (64) reported a dose-dependent effect of FA on JEG-3 cell proliferation across concentrations of 2 nM, 20 nM and 100 nM. However, their study did not include FA concentrations above 100 nM, limiting conclusions about the effects of FA excess.

In the present study, both BeWo and JEG-3 cells showed a greater magnitude of proliferative response to FA than HTR-8/SVneo cells, which is consistent with a report that BeWo cells exhibit 2–4 times higher FA uptake than HTR-8/SVneo cells (56), and may also reflect intrinsic lineage differences, as HTR-8/SVneo cells resemble differentiated extravillous trophoblasts with limited proliferative capacity, whereas BeWo and JEG-3 derive from highly proliferative choriocarcinomas (cytotrophoblast lineage). Therefore, divergent findings between studies likely reflect multiple methodological factors: the narrower FA range used by previous studies, reliance on endpoint assays rather than real-time proliferation measurements, and inherent differences in folate uptake capacity between trophoblast cell types.

Importantly, while prior studies have shown that FA can regulate trophoblast proliferation *in vitro* (56, 64), this is the first study to demonstrate a U-shaped, cell-type-specific proliferation response spanning both deficient and excess FA concentrations.

FA also non-linearly regulated the migratory and invasive capacities of HTR-8/SVneo cells (Figures 2, 3, respectively). In the present study, invasion increased under both severely deficient and excess FA conditions relative to physiological norm. Although a non-linear relationship between FA and HTR-8/SVneo invasive capacity has been reported previously (56, 65), the direction of effect differs to that observed here. Methodological differences, including use of standard FA-containing culture media (typically containing concentrations in excess of 4,000 nM), different treatment doses and different invasion assays and experimental end points, likely account for these discrepancies.

Taken together with the proliferation data, these findings suggest that trophoblast behaviour is sensitive to perturbations in FA availability, and that both deficiency and excess appear capable of disrupting the tightly regulated processes of trophoblast proliferation, migration and invasion that underpin normal placental development and function, and maternal vascular remodeling *in vivo*.

### 4.2 Placental explant proliferation and apoptosis remain unchanged by FA exposure in post-fortification tissue

In this study, proliferation and apoptosis within placental explants were not altered by FA exposure (Figure 4). Our findings are consistent with those of Ahmed et al. (56), who observed no effect of FA on proliferation or apoptosis in term placental explants. In contrast, other studies have reported a U-shaped effect of FA dose on proliferation or apoptosis in early gestation explants (66), and increased apoptosis under folate deficient conditions in human cytotrophoblasts isolated from term placentae, although excess was not assessed (67). These discrepancies may reflect several factors, including gestational age of the tissue and the nutritional background of the donor population. Specifically, studies reporting U-shaped or deficiency-induced apoptotic responses used placental tissue from non-fortified populations (66, 67), whereas studies using placentas obtained from fortified populations - including the present study and that of Ahmed et al. (56) – have not observed such effects. This suggests that chronic FA exposure *in vivo* may alter placental sensitivity to FA *in vitro.* These contextual differences underscore the importance of considering gestational age, baseline folate exposure, and population-level fortification status when interpreting *in vitro* placental studies and drawing robust conclusions.

### 4.3 Regulation of placental folate receptor and transporters

In the explant model, placental FOLR1 protein was upregulated in response to both deficient and excess FA exposure, as well as in response to moderately elevated FA levels, indicating that FOLR1 is highly sensitivity to deviations in FA concentrations (Figure 5). Additionally, PCFT abundance was also upregulated in response to 10 nM FA, a concentration representing moderate physiological deficiency, although the biological significance of this isolated PCFT response is less clear. Unlike FOLR1, which has a high binding affinity for FA and is thought to play a central role in apical folate uptake in the syncytiotrophoblast, PCFT functions via a proton-coupled transport mechanism, and may be less responsive to extracellular FA fluctuations under normal physiological conditions.

Altered FOLR1 abundance may have important implications for placental folate handling. Beyond its role in folate uptake, FOLR1 also functions as a transcription factor; therefore, changes in its abundance may influence broader placental gene expression, with potential implications for placental function, as well as maternal and fetal health. Importantly, we did not observe corresponding changes in FOLR1 or PCFT mRNA level (data not shown), suggesting that FA-mediated regulation of these proteins may occur post-transcriptionally. This highlights the importance of assessing receptor/transporter expression at the protein-level when evaluating nutrient responsiveness.

A role for FA in regulating folate receptor and transporter expression has been previously demonstrated *in vivo* (68). Mahajan et al. (68) reported that dietary FA deficiency increased *Folr1, Pcft* and *Rfc* gene expression across multiple maternal and fetal tissues (placenta, liver, brain, kidney) in mice, whereas FA over-supplementation selectively increased *Folr1* mRNA levels in the placenta, although protein abundance was not assessed. While the functional consequences of altered FOLR1 abundance remain to be determined, the current findings reinforce placental sensitivity to both low and excess FA exposure and highlight the need for further research into the impact of altered folate receptor/transporter dynamics on placental development and pregnancy health. The consequences of upregulated FOLR1 on placental FA/folate uptake and *in vivo* placental and pregnancy health are important areas for future research.

### 4.4 Altered placental hormone secretion in response to folic acid exposure

**Figure 7:**
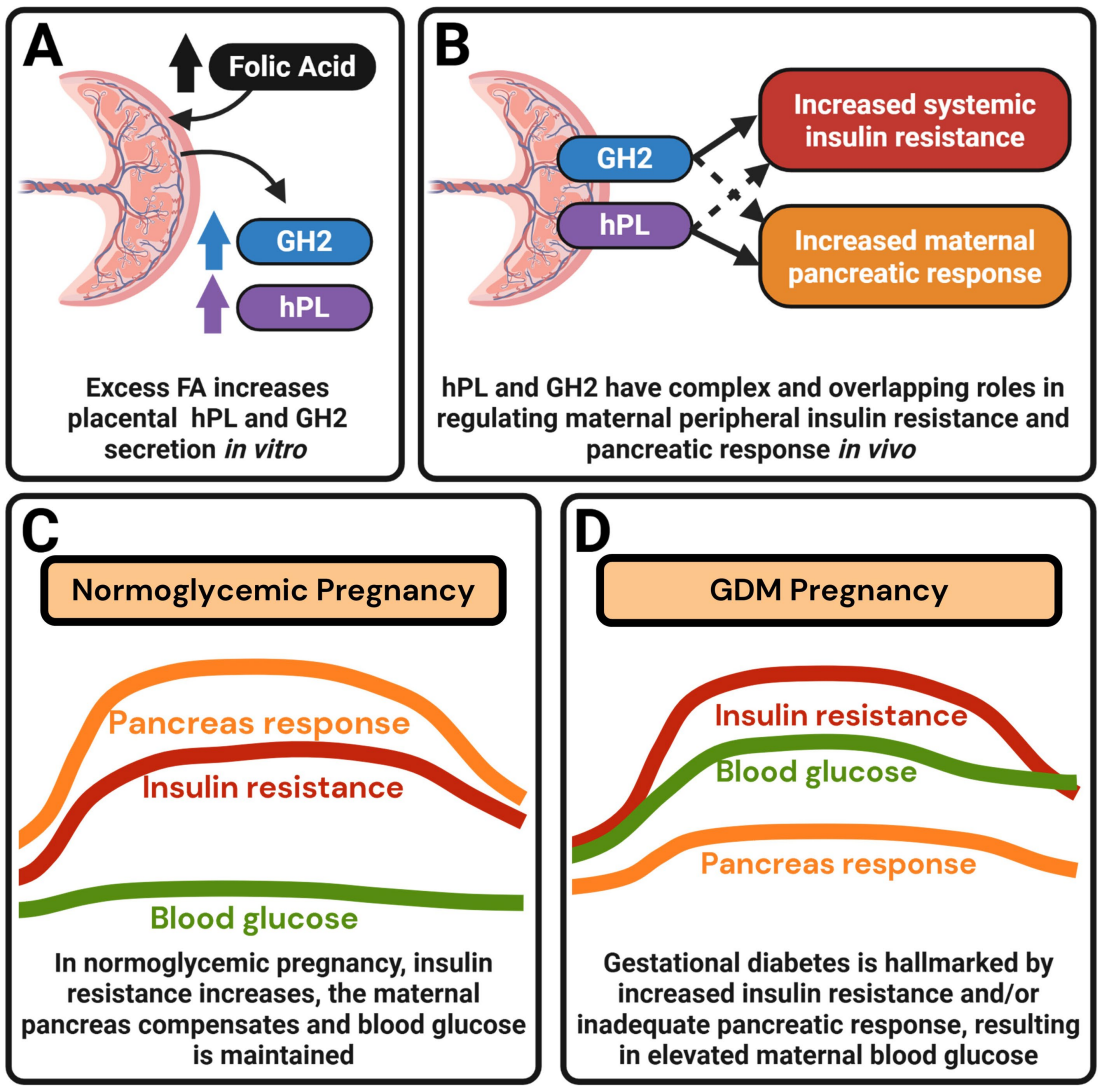
Proposed relationship between folic acid exposure, placental GH2 and hPL secretion, and maternal metabolic adaptation. **(A)** Excess folic acid increases placental GH2 and hPL secretion *in vitro*. **(B)** GH2 and hPL exert complex and overlapping roles in regulating maternal insulin resistance, glucose homeostasis, nutrient allocation, and fetal growth (47–49). **(C)** In normoglycemic pregnancy, physiological insulin resistance increases progressively, accompanied by compensatory pancreatic response to maintain euglycaemia while ensuring adequate glucose supply to the fetus. **(D)** In pregnancies complicated by gestational diabetes, insulin resistance is exaggerated and/or pancreatic response is insufficient, resulting in maternal hyperglycaemia. Solid arrows indicate primary established effects; dashed arrows indicate secondary or less well-defined roles. Figure created in BioRender. **(C,D)** adapted from Karami et al. (75).

To our knowledge, this is the first study to extensively investigate the effect of *in vitro* FA exposure on placental hormone secretion (Figure 6). We report that excess FA exposure increased secretion of hPL and GH2, and that hPL additionally exhibited a U-shaped response, with increased secretion under both deficient and excess conditions, relative to physiological norm. These findings are consistent with observational reports that women with higher circulating folate - recruited following the introduction of mandatory FA food fortification - had increased levels of hPL and GH2 in early gestation (12). In a study of pregnant adolescents, however, low folate status was associated with reduced hPL in third trimester, although excess folate status was not assessed (69). This apparent discrepancy may reflect gestational age differences: our placental explants reflect early to mid-gestation when hPL secretion is rising, whereas the adolescent study reflects term, when hPL concentrations peak and regulatory dynamics may differ. The physiological roles of GH2 and hPL in human studies are not entirely understood. GH has been shown to induce severe insulin resistance in an animal model (70), while inhibition of GH signaling improves insulin sensitivity (71). The role of hPL is less clearly defined and marked species differences in placental lactogens limit extrapolation from animal studies to humans. In cultured human islets, hPL has been shown to increase insulin secretion (72, 73), with GH exerting similar, albeit weaker, effects (72) (Figure 7). The overlap in physiological functions of hPL and GH2 is likely due to overlap in their receptor binding (74). hPL binds prolactin receptor with high affinity but can also bind growth hormone receptor with lower affinity. Conversely, GH2 primarily binds growth hormone receptor, but can also interact with the prolactin receptors (74). Given the metabolic roles of hPL and GH2 in regulating maternal insulin resistance, nutrient allocation, and fetal growth, their dysregulation by excess FA exposure may have broader implications for pregnancy health in the era of widespread food fortification and over-supplementation practices.

Other hormones measured in the present study, hCG, PAPP-A and progesterone, were not affected by FA exposure. Earlier *in vitro* studies using JEG-3 (64) and BeWo cells (56), as well as term placental explants (56), similarly reported no effect of FA on hCG or progesterone secretion. To our knowledge, no prior studies have examined FA regulation of placental PAPP-A secretion, although higher circulating PAPP-A concentrations have been associated with low folate status in women (69).

### 4.5 Study summary, clinical implications and future directions

This study provides a comprehensive assessment of the effects of FA, ranging from severe acute deficiency to physiological excess, on key aspects of placental function. Trophoblast cell lines demonstrated marked responsiveness to FA, with consistent parallels observed between the effects of deficiency and excess across multiple functional endpoints. In human placental explants derived from first and second trimester pregnancies post FA-fortification, FA exposure did not alter proliferation, apoptosis, nor secretion of hCG, PAPP-A and progesterone, However, significant changes were observed in the secretion of GH2 and hPL, as well as in FOLR1 protein abundance, suggesting selective sensitivity of placental endocrine and folate transport pathways to FA exposure. These findings provide novel evidence that FA may modulate specific placental functions, not only under conditions of deficiency, as previously emphasised, but also in the context of excess. Given the critical role that these hormones play in modulating maternal insulin resistance during pregnancy (Figure 7), and in the context of widespread FA food fortification and high compliance to prenatal supplementation (often with doses exceeding the recommended 400 μg/day), these findings are highly significant. Urgent studies are needed to evaluate the impact of elevated FA exposure on placental endocrine function, particularly its potential to disrupt metabolic adaptations of pregnancy in ways that may impair maternal glucose handling and increase susceptibility to GDM.

## Data Availability

The raw data supporting the conclusions of this article will be made available by the authors, without undue reservation.
